# The *Candida albicans* Pho4 Transcription Factor Mediates Susceptibility to Stress and Influences Fitness in a Mouse Commensalism Model

**DOI:** 10.3389/fmicb.2016.01062

**Published:** 2016-07-07

**Authors:** Verónica Urrialde, Daniel Prieto, Jesús Pla, Rebeca Alonso-Monge

**Affiliations:** Departamento de Microbiología II, Facultad de Farmacia, Universidad Complutense de MadridMadrid, Spain

**Keywords:** *Candida albicans*, virulence, stress response, transcription factor, fitness, commensalism

## Abstract

The Pho4 transcription factor is required for growth under low environmental phosphate concentrations in *Saccharomyces cerevisiae*. A characterization of *Candida albicans pho4* mutants revealed that these cells are more susceptible to both osmotic and oxidative stress and that this effect is diminished in the presence of 5% CO_2_ or anaerobiosis, reflecting the relevance of oxygen metabolism in the Pho4-mediated response. A *pho4* mutant was as virulent as wild type strain when assayed in the *Galleria mellonella* infection model and was even more resistant to murine macrophages in *ex vivo* killing assays. The lack of Pho4 neither impairs the ability to colonize the murine gut nor alters the localization in the gastrointestinal tract. However, we found that Pho4 influenced the colonization of *C. albicans* in the mouse gut in competition assays; *pho4* mutants were unable to attain high colonization levels when inoculated simultaneously with an isogenic wild type strain. Moreover, *pho4* mutants displayed a reduced adherence to the intestinal mucosa in a competitive *ex vivo* assays with wild type cells. *In vitro* competitive assays also revealed defects in fitness for this mutant compared to the wild type strain. Thus, Pho4, a transcription factor involved in phosphate metabolism, is required for adaptation to stress and fitness in *C. albicans*.

## Introduction

*Candida albicans* is an opportunistic pathogen that is a commensal of the skin and mucosal surfaces of humans. This fungus can cause diverse infections, known as candidiasis that range from superficial to systemic, being a prominent cause of nosocomial systemic infections in developed countries (Pfaller and Diekema, 2007, 2010). As an opportunistic pathogen, *C. albicans* switches from commensal to pathogen and in this process, fungal cells have to face different pHs, nutritional environments and osmotic challenges. *C. albicans* has therefore to adapt to different physiological niches assimilating diverse nutrients, tolerating diverse host temperatures and facing diverse stresses generated within the host. Remarkably, they also have to compete with other members of the microbiota and host immune defenses. Its ability to cause infection, therefore, largely depends not only on fungal virulence factors but on fitness attributes (reviewed by Mayer et al., 2013). We will consider fitness in this context as all functions required for microbial growth that are relevant for the virulence of the microbe, although they may not necessarily be components that physically interact with the host cells (Navarro-García et al., 2001).

The relevance of metabolic adaptation has been analyzed in terms of carbon sources but can be extended to others nutrients such as nitrogen, oxygen, phosphorus, or micronutrients (i.e., iron). Phosphorus, as inorganic phosphate (Pi), is an essential component of various structural biomolecules and participates in cellular energy storage. The maintenance of phosphate homeostasis is therefore crucial for all living organisms. Ascomycetes have a pathway (named PHO) which monitors phosphate cytoplasmic levels and controls the expression of genes involved in phosphate uptake and sensing from external sources as well as the mobilization of internal phosphate stock (Tomar and Sinha, 2014). The response to Pi concentration changes is well-documented in *Saccharomyces cerevisiae*. When the surrounding Pi is high, the transcription factor Pho4 is hyper-phosphorylated by the Pho85-Pho80 complex being excluded from the nucleus that switches off the pathway. In these conditions, the low affinity phosphate transporters Pho87 and Pho90 are upregulated and mediate the uptake of external phosphate. The excess of phosphate is stored in vacuoles as PolyP (polyphosphate). When the Pi is low in the environment, PolyP is mobilized by polyphosphatases that work independently of the PHO pathway. If this reserve is not enough, budding yeast turn on the PHO regulatory system: Pho81 represses the Pho85-Pho80 complex and Pho4 is hypo-phosphorylated, remaining in the nucleus where it induces the transcription of high affinity phosphate transporters such as Pho84 and Pho89 and secretory phosphatases such as Pho5, Pho11, and Pho12 among others genes (Tomar and Sinha, 2014).

In the *C. albicans* genome, 24 genes homologous to the PHO pathway genes from *S. cerevisiae* have been identified. Despite this fact, little is known about their function in *C. albicans* physiology and pathogenicity. The lack of the transcription factor Pho4 sensitizes *C. albicans* to phosphate limitation, inducing extensive filaments in low phosphate conditions (Romanowski et al., 2012). The intestinal tract of critically ill patients displays phosphate depletion and accordingly, clinical isolates from these patients show marked responsiveness to phosphate limitation which may represent a fitness adaptation to the complex and nutrient scarce environment typical of the gut of these patients (Romanowski et al., 2012). These strains display an enhanced virulence, resulting in host death in animal models, although the molecular bases for this phenotype remain unknown.

Given that *C. albicans* needs to cope with different stresses during growth as a commensal compared to growth as a pathogen and that the transcription factors involved in this process are not well-characterized in this yeast, we performed a screening searching uncharacterized transcription factors involved in stress responses. Unexpectedly, the lack of *PHO4* rendered mutants susceptible to both osmotic and oxidative stress. This transcription factor has been recently implicated in the response to inorganic arsenic compounds (Urrialde et al., 2015) and therefore, it must play additional roles to Pi homeostasis. Here, we have aimed to analyze the role of *C. albicans* Pho4 in the adaptation to stress and define its role in the virulence and adaptation to comensalism of this fungus.

## Materials and Methods

### Strains and Growth Conditions

Yeast strains used are listed on **Table 1**. The *C. albicans* wild type, SFY87 and the *pho4* defective mutant SFY5 are described (Vandeputte et al., 2012) and available at the Fungal Genomic Stock Center^1^. The *PHO4* reintegrant designates a *pho4* mutant where the C-terminal myc tagged version of *PHO4* under the control of the repressible tetracycline promoter OP4 was integrated at the *ADH1* locus of the *C. albicans* genome (Urrialde et al., 2015). To label the *C. albicans* strains, pNIM1R-GFP and pNIN1R-dTOM2 plasmids carrying, respectively, the GFP and dTOM2 fluorescent proteins under the control of the repressible tetracycline promoter OP4 (Prieto et al., 2014) were digested with *Kpn*I- *Ksp*I and integrated at the *ADH1 locus*. The generated strains were VUR1 (the wild type strain tagged with GFP), VUR2 (the wild type tagged with the red dTOM2 version), and VUR10 (dTOM2 tagged *pho4* mutant; **Table 1**).

**Table 1:** Strains used in this study.

Strain	Genotype	Nomenclature in text and figures	Source
SFY87	*ura3::imm434/ura3::imm434* *iro1/iro1::imm434* *his1::hisG/his1::hisG* *arg4/arg4 RPS1/rps1::CIp30* (*URA3, HIS1, ARG4*)	wt	Fungal Genetic Stock Center (http://www.fgsc.net) (Dhamgaye et al., 2012)
SFY5	*ura3::imm434/ura3::imm434* *iro1/iro1::imm434* *his1::hisG/his1::hisG arg4/arg4* mutant generated by TIGR transposon collection	*pho4*	Fungal Genetic Stock Center (http://www.fgsc.net)
SFY5-R	*pho4 ARD1/ard1::tTA Ptet-PHO4myc-SAT1*	*PHO4^reint^*	Urrialde et al., 2015
VUR1	SFY87 *ADH1/adh1::tTA Ptet -dTOM2-SAT1*	SFY87-dTOM2 wt-dTOM2	This work
VUR2	SFY87 *ADH1/adh1::rtTA Ptet -GFP-SAT1*	SFY87-GFP wt-GFP	This work
VUR10	*pho4 ADH1/adh1::tTA Ptet -dTOM2-SAT1*	*pho4*-dTOM2	This work

Yeast strains were routinely grown at 37°C in YPD medium (1% yeast extract, 2% peptone, and 2% glucose; all reagents from Panreac). In those experiments that require to differentiate two *C. albicans* populations (*C. albicans* labeled with the expression fluorescence proteins) cultures were spread on SD (2% glucose, 0.67% yeast nitrogen base) plus amino acids. When *C. albicans* was taken from stools or organs it was spread on SD medium supplemented with chloramphenicol (10 mg/L).

The influence of atmosphere surrounding was analyzed either incubating the plates in an incubator designed for cell culture or in an anaerobic chamber. The cell culture incubator was programmed at 37°C, 80% humidity and 5% CO_2_ in the presence of atmospheric O_2_. Hypoxia was achieved using an anaerobic chamber and a commercial system that ensures less than 0.1% O_2_ in 2.5 h and more than 15% CO_2_ (GENBox anaer, BioMérieux).

### Stress Susceptibility Screening

A 241 transcription factor knock-out library provided by Dr. D. Sanglard was tested to identify mutants defective in osmotic and/or oxidative stress adaptation. *C. albicans* strains were grown overnight in YPD at 37°C, then, O.D. was measured to enable equalize yeast suspensions to 0.8 O.D. in 96-wells microtiter plates. From the original plate 10-fold dilutions were performed in different plates and spotted on YPD plates supplemented with 2 or 3 mM diamide, 150 or 200 μM menadione, 4,5 or 7 mM hydrogen peroxide, 1 or 1.5 M NaCl or 1 or 2 M sorbitol. Mutants were analyzed in the same plate than its parental strain together with a sensitive mutant as control. Plates were incubated at 37°C for 24–48 h and, then, visualized to detect mutants displaying growth defects on any of the analyzed conditions. Those mutants that displayed any phenotype were checked again including others clones if they were available in the knock-out collection. If susceptibility differences were detected among clones, mutant was excluded from the analyses.

### Compounds Susceptibility Assays

Drop tests were performed by spotting 10-fold serial dilutions of cells onto YPD plates supplemented with the compounds and concentration indicated. Plates were incubated at 37°C. Kinetics of susceptibility to GSNO (ENZO Life sciences) was measured using exponentially growing cells: 10^7^ cells were transferred to a 1.5 mL tube containing 1 mL of YPD broth and the nitric oxide generator was added to the final concentrations indicated. Tubes were incubated at 37°C with shaking and 5 μL samples were collected at different time points and cultured in YPD or SD agar plates. The plates were incubated for 24 h at 37°C and colony forming units (CFUs) were counted. Relative viability was calculated as percentage of CFUs at different point versus CFUs before adding GSNO. Two-way ANOVA analyses were used to detect significant differences.

### Virulence Assays in *Galleria mellonella*

SFY87 (wt) and *pho4* mutant were grown overnight at 37°C; cells were recovered by a low speed centrifugation and were washed twice in phosphate buffer saline (PBS). Then, cells were resuspended in PBS and cell number was determined using a Neubauer chamber; 10^6^ cells in 10 μL were injected directly into the hemocoel at the last left pro-leg using a Hamilton syringe. Each infection group contained 20 larvae of approximately 400–500 mg weight. Two group controls were used: larvae injected with PBS and larvae not inoculated. Larvae were maintained at 37°C in darkness. Survival was monitored for 9 days after infection. Kaplan–Meier survival curves are shown and Log-rank (Mantel–Cox) test statistical analyses were performed.

### Murine Intestinal Commensalism Model

All experiments involving animals performed in this work were carried out in strict accordance with the regulations in the “Real Decreto 1201/2005, BOE 252” for the Care and Use of Laboratory Animals of the “Ministerio de la Presidencia,” Spain. The protocol used in the commensalism model was approved by the Animal Experimentation Committee of the University Complutense of Madrid (CEA 25/2012, BIO2012-31839-1) and Comunidad de Madrid according to Artículo 34 del RD 53/2013. All efforts were made to minimize suffering, even though the treatments did not result in disease in the animals. The number of animals used in the experimentation was minimized for ethical reasons.

Female mice C57BL/6 were obtained from Harlan Laboratories, Inc. (Italy) and used within an age of 7 to 10 weeks-old. Mice housing and other non-invasive procedures took place in the animal facility from the Medical School of the Universidad Complutense de Madrid.

The protocol for studying commensal colonization used in this work has been described previously (Prieto et al., 2014). Briefly, after 4 days of antibiotic pre-treatment (2 mg/mL streptomycin, 1 mg/mL bacitracin, and 0.1 mg/mL gentamycin), 10^7^ *C. albicans* cells were inoculated in a single gavage. Stool samples obtained every 2–4 days, they were homogenized in PBS and cultured in SD plates to determine CFUs per gram. To analyze *C. albicans* loads in different portions of the gastrointestinal tract, mice were sacrificed and samples from stomach, cecum, small and large intestine were aseptically separated, homogenized and diluted in sterile PBS and cultured in SD plates.

### Adhesion Assays

To analyze the adhesion capacity of the *pho4* mutant to intestinal mucosa we proceed as previously described (Prieto et al., 2014). A 1 cm-piece of the large intestine (from a recently euthanatized mouse), was opened, washed and placed in a 4 mm-diameter methacrylate chamber, which was filled with RPMI medium pre-warmed at 37°C. Then, *C. albicans* strains from overnight YPD cultures were mixed (1:1) and adjusted to 2.5 × 10^7^ cells/mL concentration in serum-free RPMI medium. 40 μL of this suspension (10^6^ yeast cells) were placed in the lumen side from the colonic tissue and incubated for 2.5 h at 37°C. Then, the piece of intestine was carefully washed with sterile PBS twice and mechanically disaggregated. Cells of *C. albicans* from the latter fraction were considered as adhered and further analyzed by CFUs determination. Adhesion Relative Index was calculated by dividing the percentage of adhered cells from (SFY87 or *pho4*)-dTOM2 strains recovered by their percentage in the inoculum.

Adhesion to plastic was performed in 24-well flat bottom plates for culture cells. 500 cells were added to each well in RPMI 1640 medium and allowed to adhere for 30 min, 1 and 2 h. Medium carrying non-adhered cells was spread on YPD (when single cultures were analyzed) or SD media (for mixed cultures) for CFUs count. Adhered cells were mechanically removed and spread on YPD or SD media for CFUs count. Single culture adherence was expressed as percentage of adhesion [adhered cells^∗^100/(adhered cells + non-adhered cells)]. Mixed cultures adhesion was expressed as the ratio of the Adherence Relative Index (ARI) of adhered cells to the ARI of non-adhered cells (ARI^a^/ARI^na^) of two independent experiments; each experiment was performed in duplicate. ARI was calculated as above mentioned, that is dividing the percentage of adhered cells from strains labeled with dTOM2 at each time points by their percentage in the inoculum.

### Mammalian Cell Culture and Infections Assays

The RAW264.7 cell line was obtained from the ATCC (American Type Culture Collection) and was maintained in RPMI 1640 (Gibco) supplemented with 10% heat inactivated fetal bovine serum (Gibco), glutamine (Gibco) and 1% streptomycin/penicillin (Gibco) at 37°C in 5% CO_2_. For harvesting, cells were centrifuged at 2000 r.p.m, washed with PBS and resuspended in completed RPMI at the required final concentration. The cell population was counted by trypan blue dye exclusion. Peritoneal macrophages were obtained from mice. 1 mL 3% thioglycolate was injected in the peritoneal cavity of three mice, after 3 days. 10 mL completed RPMI were injected in order to recover the immune cells localized at the peritoneal cavity. Cell number (assumed to be mainly macrophages) was determined using a Neubauer chamber and split on 24-well plate at the required final concentration. Cells were incubated in a cell culture incubator for 24 h before the killing assay.

For killing assays, phagocytes and *C. albicans* strains were suspended in completed RPMI 1640 medium at a cell ratio of 1:40 (yeasts: phagocytes). The cultures were incubated at 37°C in 5% CO_2_ for 2 h. At the end of the experiment SDS (dodecyl sulfate sodium salt) was added to a final concentration of 0.05% to lyse mammalian cells. Assays were done a total of four times, and the percentage of viability of each strain was expressed as the percent reduction of CFUs from phagocyte–yeasts co-cultures versus *C. albicans* suspension before incubation according to the formula 100-[(CFUs inoculum-CFUs co-cultures)/(CFUs inoculum)] X 100. Differences between two groups were calculated using Student’s two-tailed unpaired *t*-test.

## Results

### *pho4* Mutants Are Sensitive to Both Osmotic and Oxidative Stress

**FIGURE 1:**
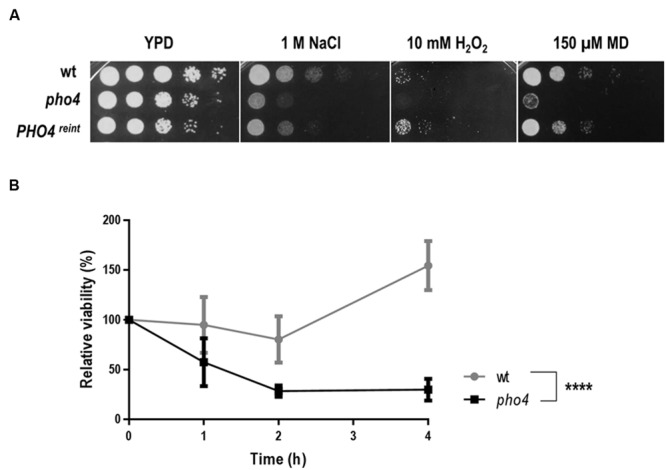
**Pho4 mediates the response to different stresses. (A)** 10-fold cell dilutions of the indicated strains were spotted on YPD plates supplemented with indicated compounds. Plates were incubated at 37°C for 18 h. MD means menadione. **(B)** Liquid YPD cell suspensions were exposed to 0.6 mM GSNO, incubated at 37°C and samples collected at different time points. Cells were spread on SD plates and viable cells were count as CFUs. Relative viability was expressed as percentage of CFUs at different times per CFUs prior GSNO addition. Two-way ANOVA test was performed to evidence statistical differences.

In order to identify transcription factors involved in the response to different types of stress, a 241 transcription factor knock out library from *C. albicans* (Vandeputte et al., 2012) was assayed in drop tests on YPD plates supplemented with different oxidants (diamide, menadione, hydrogen peroxide) or osmotic stress generators (NaCl or sorbitol). Plates were incubated for 24–48 h and visually checked for resistance or sensitivity. **Table 2** summarizes the transcription factor mutants and the phenotypes detected in the screening. 11 mutants displayed susceptibility to any oxidative agent, of these, 2 transcription factors have been previously implicated in oxidative stress response, Cap1 and Skn7 (Alarco and Raymond, 1999; Singh et al., 2004). The susceptibility to osmotic and/or oxidative stress was confirmed and shown in **Supplementary Figure S1**. Only one mutant, *pho4*, displayed susceptibility to both oxidative and osmotic stress (**Supplementary Figure S1** and **Figure 1A**). Pho4 is a bHLH transcription factor of the myc-family previously involved in the response to phosphate limitation and arsenic compounds (Romanowski et al., 2012; Urrialde et al., 2015). Although, the *pho4* mutant seemed to grow slower on the YPD control plate, no significant differences (as determined using linear regression analyses) between SFY87 and the *pho4* mutant strains were detected when cultures were grown in liquid medium (**Supplementary Figure S2**). Reintegration of the *PHO4* ORF under the tightly regulated Tet-OFF promoter reverted, as expected, the sensitivity to both stresses, osmotic and oxidative (**Figure 1A**). We also tested the susceptibility to nitrosative stress, given its importance in the killing mechanism of phagocytic cells (Rementería et al., 1995; Vazquez-Torres and Balish, 1997; Brown et al., 2009). In this case, a suspension of 10^7^ cells was challenged with 0.6 mM GSNO, a nitric oxide generator, and samples were collected 1, 2, and 4 h later. Cells were spread on YPD plates and CFUs were determined. As shown in **Figure 1B**, the viability decreased in both strains after 2 h of incubation (∼80% in the wild type and ∼28% in the *pho4* mutant) indicating than GSNO is toxic for *C. albicans* strains. However, the differences between the *pho4* mutant and the wild type strain were significant (**Figure 1B**). After 4 h of incubation in the presence of GSNO, the wild type strain started to increase in CFUs count (154.5% relative viability) suggesting that either the GSNO dropped or the wild type strain was able to adapt and detoxify RNS. In contrast, the *pho4* mutant retained a relative low viability (29.9%) suggesting that the lack of Pho4 rendered cells unable to tolerate nitric oxide; thus, Pho4 is also involved in nitrosative stress resistance.

**Table 2:** Transcription factor mutants that display increased susceptibility to the indicated stress.

	Osmotic stress		Oxidative stress		
	NaCl (1, 1.5 M)	Sorbitol (1, 2 M)	H_2_O_2_ (4, 5, 7 mM)	Diamide (1.5, 3 mM)	Menadione (150, 200 μM)
*pho4*	+++	+++	+++	++	+++
*skn7*	=	=	+++	=	=
*upc2*	=	=	++	++	=
*glo3*	=	=	++	++	=
*zcf13*	=	=	++	++	=
*orf19.2260*	=	=	+++	++	++
*mig1*	=	=	=	++	+
*ada2*	=	=	+	+	+
*rlm1*	=	=	=	++	=
*wor3*	=	=	=	+++	+++
*cap1*	=	=	+++	+++	+++

*+++: very sensitive; ++: sensitive; +: slightly sensitive; =: similar to wild type.*

### Pho4 Is Neither Required for Virulence in a Non-vertebrate Model nor Phagocytes Resistance

**FIGURE 2:**
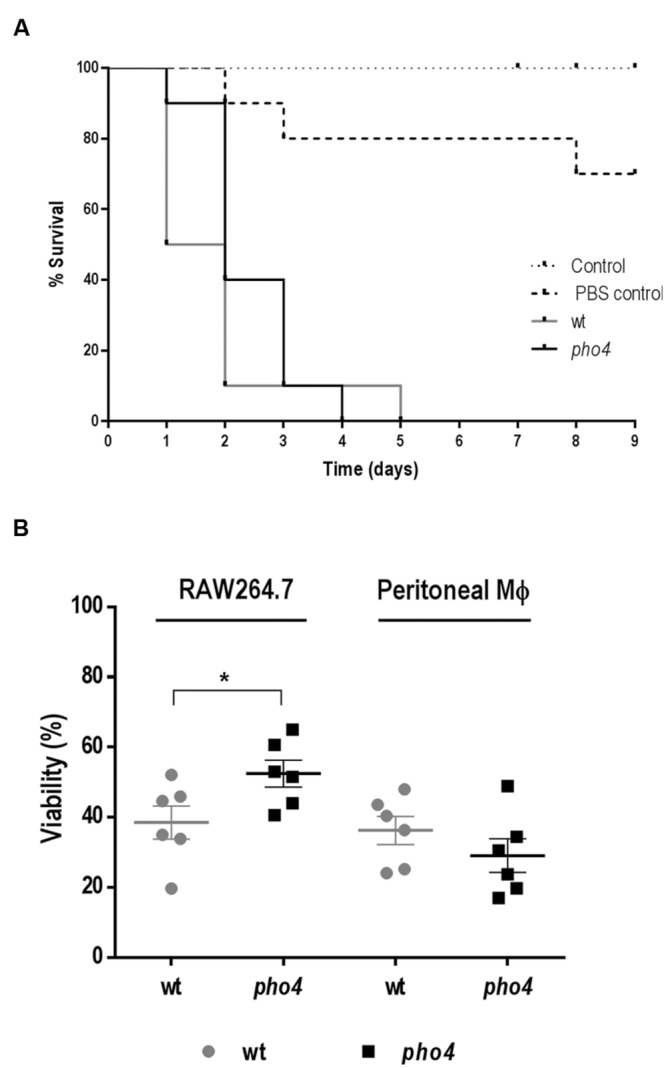
**Role of Pho4 in virulence. (A)** Survival curve of *Galleria mellonella* larvae. 10^6^ *Candida albicans* cells were injected at the last left pro-leg of G. mellonella larvae and survival of the insect was followed in time. Control means *G. mellonella* larvae no inoculated and control PBS indicates larvae inoculated with PBS as stress control. Kaplan–Meier survival curves are shown. Log-rank (Mantel–Cox) test displayed no significant differences between wild type and *pho4* mutant (*p*-value 0.2758). **(B)** Relative viability of *C. albicans* strains in the presence of murine macrophage cell line (RAW 264.7) or peritoneal extracted murine macrophages (Peritoneal MΦ). The percentage of viability was quantified after 2 h of co-incubation at a cell ratio 1:40. The graph shows six independent experiments. The Error bar is the standard error of the mean (SEM). Statistical differences among two groups were calculated using Student’s two-tailed unpaired *t*-test ^∗^*p* < 0.05 (*p* < 0.0444 for RAW264.7 and *p* < 0.3457 for peritoneal macrophages).

Since *pho4* cells were susceptible to stresses operating in immune cells, we wondered if Pho4 would be required during infection. To address this question, we first used the *Galleria mellonella* model. This alternative non-vertebrate model of infection has certain ethical and technical advantages compared to the commonly used murine systemic model (reviewed by Fuchs et al., 2010; Junqueira, 2012; Jacobsen, 2014). PBS injected and non-inoculated (negative) larvae were used as controls (**Figure 2A**). All larvae inoculated with *C. albicans* died within 5 days after challenge but Log-rank (Mantel–Cox) test statistical analyses revealed no significant differences between wild type and *pho4* mutant strains. Given the immunologic differences between this model and mammalian’s, we also tested the susceptibility to mammalian phagocytes using two different *ex vivo* assays. Killing assays were performed using activated macrophages isolated from the murine peritoneal cavity and the murine macrophage cell line RAW 264.7. When activated macrophages were co-incubated with *C. albicans* strains no significant differences were observed regarding viability between *pho4* mutant [29.1% ± 4.8 SEM (standard error of the mean)] and its parental strain (36.2% ± 4.0 SEM). However, in the presence of the RAW 264.7 cell line, the results were different and statistically significant differences were observed between the wild type (38.5% ± 4.7 SEM) and the *pho4* mutant (52.5% ± 3.8 SEM) in co-infection assays. With RAW 264.7 macrophages, *C. albicans pho4* cells displayed a clear increase in viability compared to peritoneal macrophages (**Figure 2B**). Since the results depended on the type of macrophages used in the study, another phagocyte model was tested. When the promyelocytic human cell line HL-60 differentiated to PMNs was used, no significant differences were detected (**Supplementary Figure S3**). Collectively, these data indicate that Pho4 is neither required for virulence nor survival in phagocytes.

### Pho4 Does Not Impair the Colonization in the Intestinal Proximal Tract

**FIGURE 3:**
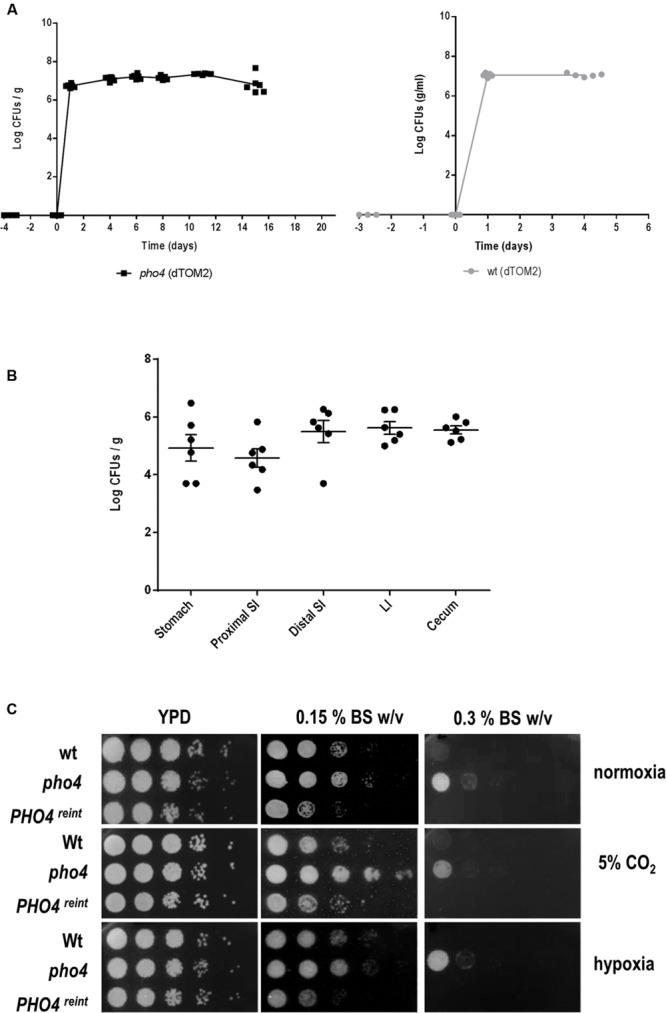
**Role of Pho4 in murine gut colonization. (A)** The *pho4* mutant (left graph) and the wild type (right graph) strains tagged with the dTOM2 reporter gene were inoculated by gavage in antibiotic treated C57BL/6 mice. *C. albicans* colonization was followed in time by counting CFUs from stools. Graph represents Log CFUs/g of stools versus time. Each square represent a single mouse (n = 6). **(B)** Intestine from mice colonized with pho4-dTOM2 mutant were split on stomach, proximal small intestine (SI), distal small intestine, large intestine (LI) and cecum. Samples were processed and *C. albicans* colonies counted. Each single independent value is represented and the mean ± SEM from six mice. **(C)** Drop test was performed on YPD plates supplemented with Bile Salts (BS) to analyze the indicated strains susceptibility. Plates were incubated under the specified conditions at 37°C for 24 h (normoxia and 5% supplemented CO_2_ atmosphere) or 48 h (for plates incubated under hypoxia).

Certain mutants defective in stress signaling have been shown to be impaired in colonizing the murine intestinal tract (Prieto et al., 2014). We therefore tested if the transcription factor Pho4 played also a role in commensalism using this gastrointestinal model of colonization. A *pho4* mutant expressing a red fluorescent protein optimized for *C. albicans* expression, dTOM2 (Prieto et al., 2014), was inoculated intragastrically in C57BL/6 mice. The intestinal tract colonization was followed over time by counting CFUs from stools. The *pho4* mutant was able to establish a normal commensal colonization, attaining high fungal levels in the range of 10^7^ CFU/g and similar to its parental strain (SFY87; **Figure 3A**). Additionally, the intestinal content was analyzed post mortem at the end of the experiment (20 days). The *pho4* mutant was able to colonize the whole intestine, but its levels were reduced in the stomach and proximal small intestine (**Figure 3B**) as occurs in this model of colonization (Bendel et al., 2002). CFUs oscillated among 4.9 log CFUs per gram ± 0.45 SEM at the distal small intestine, 5.5 log CFUs per gram ± 0.4 SEM at large intestine and 5.6 log CFUs per gram ± 0.3 SEM at the cecum. As the proximal small intestine is especially rich in bile salts, we checked the susceptibility *in vitro* to this substance on solid medium. Remarkably, we found that the *pho4* mutant tolerates bile salts even better than the wild type (**Figure 3C**) under different environmental conditions, indicating that this was not the cause of reduced colonization in the small intestine. We conclude from this set of experiments that Pho4 is dispensable for colonization of *C. albicans* in the murine intestine.

### Stress Resistance Mediated by Pho4 Depends on O_2_ Availability

**FIGURE 4:**
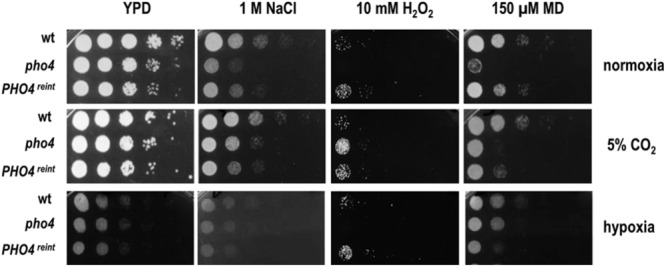
**Influence of the atmosphere in the susceptibility of *pho4* to stress**. Indicated *C. albicans* strains were spotted on YPD plates supplemented with the indicated compounds and incubated at 37°C for 24 h (normoxia and 5% supplemented CO_2_ atmosphere) or 48 h (for plates incubated under hypoxia). MD means menadione.

The absence of Pho4 did not show any impairment in virulence or intestinal tract colonization in spite of being more susceptible to ROS and RNS *in vitro*. However, the mouse gut environment represents a strikingly different condition compared to classic *in vitro* incubation in several aspects. One of them is oxygen availability, which takes place in normoxia when growing *in vitro* or in a CO_2_ atmosphere, when doing interaction studies with mammalian cell; however, these differ from the scarce oxygen availability (almost anaerobiosis) found in the gut. We therefore re-analyzed the susceptibility to different stresses in the presence or absence of O_2_ and CO_2_. Interestingly, when the plates were incubated at 37°C in normoxia with 5% CO_2_ (the conditions that are routinely used to grow mammalian cells and perform host interaction studies) *pho4* cells clearly improved their growth in the presence of NaCl and H_2_O_2_, and also grew slightly better in the presence of menadione compared to the wild type strain (**Figure 4**). Under anaerobic conditions, all strains grew slower and the *pho4* mutant behaved essentially as a wild type on 1 M NaCl or menadione plates (**Figure 4**). These results indicate that the function of Pho4 is dependent on the oxygen availability, being less important in those conditions were CO_2_ is present or O_2_ is not accessible. These observations are coherent with our previous results regarding susceptibility to phagocytes. Furthermore, the surrounding atmosphere plays no significant effect on bile salts resistance (**Figure 3C**), indicating that this *pho4* mutant phenotype does not depend on O_2_ availability.

### The Lack of Pho4 Impairs Fitness in *C. albicans*

**FIGURE 5:**
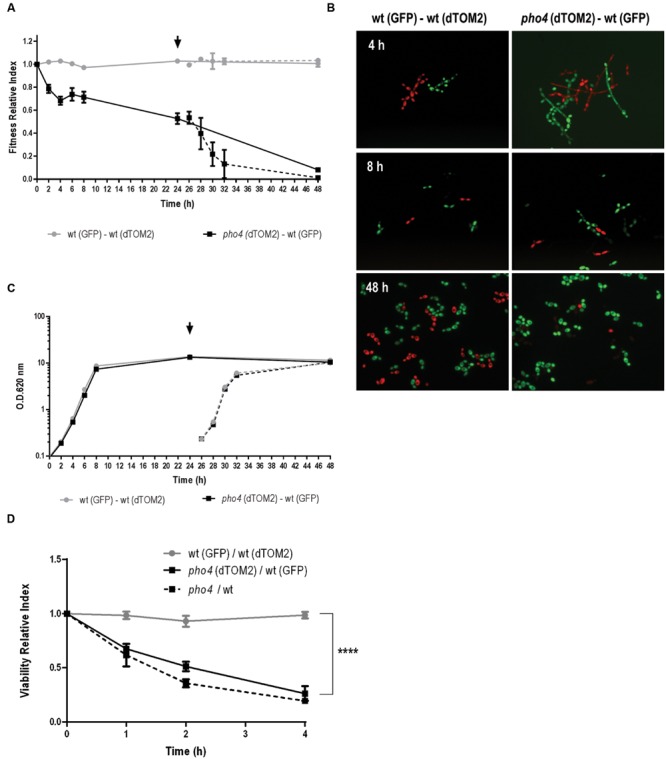
***pho4* mutant is replaced by wild type strain in mixed cultures. (A)** Exponentially growing SFY87-GFP/SFY87-dTOM2 (gray circles) and SFY87-GFP/*pho4*-dTOM2 (black square) strains were mixed to equal amount to 0.2 O.D. in YPD medium. Samples were collected at different time points and spread on SD plates to differentiate and count both types of colonies. After 24 h of growth a part of the culture was refreshed to new pre-warmed YPD medium and followed on time (dashed line). Fitness Relative Index was represented as function of time. **(B)** Mixed cultures were observed under fluorescent microscopy and representative samples are shown. **(C)** In parallel, cultures growth was determined as O.D.: (A_260__nm_) and represented as function of time. **(D)** Balanced SFY87-GFP/SFY87-dTOM2 (gray circles), SFY87-GFP/*pho4*-dTOM2 (black square) mixed cell suspensions were challenged with 0.6 mM GSNO and incubated at 37°C. Samples were taken at different time points and spread on SD plates for CFUs count. Viability Relative Index was calculated as the ration between percentage of viable cells and the percentage of cells in the initial inoculum. Data from single cultures of wild type and *pho4* mutant exposed to the same treatment were similarly analyzed. *pho4*/wt represent the Viability Relative Index calculated from single cultures. The graph shows the media ± SEM of three independent experiments. Statistical significance was determined using the Sidak-Bonferroni method, ^∗∗∗^p < 0.001.

Given that differences between a *pho4* mutant and wild type are not clearly observed under hypoxia or 5% CO_2_ atmosphere, we asked if the lack of Pho4 could provide an evolutionary advantage for *C. albicans*. For this purpose, we analyzed the behavior of *pho4* mutant and its wild type strain (SFY87) in a competitive culture assay. Both strains were labeled with a genetic system that allows tracing and differentiating two populations (Prieto et al., 2014; Prieto and Pla, 2015). The labeled strains were grown to stationary phase in YPD and then mixed to 0.2 O.D. The mixed cultures were incubated in agitation at 37°C and samples were analyzed via CFUs counting for assessing the relative percentage of each population. The relative quantification of wt-GFP versus *pho4*-dTOM2 was expressed as Fitness Relative Index and represented at different time points (**Figure 5A**, black line). As shown in **Figure 5A** the relative amount of *pho4*-dTOM2 strain sharply decreased with time and the *pho4* mutant was almost completely replaced with the wt-GFP after 48 h of co-incubation. In parallel, a control culture was followed in time where the wild type strain labeled with GFP or dTOM2 fluorescent proteins were mixed to equal amount (**Figure 5A**, gray line). The Fitness Relative Index for wild type mixed culture was, as expected, around 1 indicating that the label has not effect on growth. Mixed cultures were observed under fluorescent microscopy: mixed *pho4*-dTOM2/ wt-GFP culture grew as filaments when culture was inoculated in fresh medium (see **Figure 5B**, 4 h right panel). After 8 h, cultures grew mainly as yeast and after 48 h the red fluorescence decreased drastically in the *pho4*-dTOM2/wt-GFP mixed culture indicating that *pho4* mutant was replaced by the wild type strain. Green and red fluorescence signals were similar for wt-GFP/wt-dTOM2 mixed cultures.

In order to discard an effect of the medium resulting from the co-culture, after 24 h of incubation samples were taken and re-inoculated in fresh pre-warmed YPD medium. These refreshed cultures were followed in time and samples spread on SD plates at different time points. The Fitness Relative Index after refreshing the cultures displayed the same pattern than in the pre-inoculum and red colonies (*pho4*-dTOM2) decreased significantly compared to colonies of wt-GFP (**Figure 5A**, dashed lines). The control culture (wt-GFP/wt-dTOM2) maintained a similar proportion of both labels (Fitness Relative Index ∼1, gray dotted line) indicating that the loss of red colonies was not due to the dTOM2 label but to the mutant. A standard growth curve was performed in parallel (**Figure 5C**). The graph showed that both mixed cultures (wt-GFP/wt-dTOM2 and *pho4*-dTOM2/wt-GFP) grew similarly since both growth curves overlapped. Single cultures were also analyzed. The growth curve of single cultures overlapped with the mixed growth curves. No differences were detected between strains, labeled or not, or grown in complex (YPD) or in defined (SD) media (**Supplementary Figure S2**). The lack of Pho4 did not alter significantly the growth rate or the final (overnight) O.D. in liquid media in single culture. We therefore conclude that *pho4* mutants are replaced by wild type when grown in liquid mixed cultures.

We also determined if the co-culture of strains had any effect on stress susceptibility. To achieve this goal, the susceptibility to nitrosative stress in a mixed culture was determined. As shown in **Figure 5D**, a suspension of balance mix of wt-GFP and *pho4*-dTOM2 or wt-GFP and wt-dTOM2 (as control) were challenged with 0.6 mM GSNO which spontaneously generates nitric oxide. The *pho4* mutant lost viability significantly faster than wild type but comparable to the same strains in single cultures (named *pho4*/wt in **Figure 5D**). This observation indicates that the *pho4* mutant was similarly sensitive to nitrosative stress either in mixed or single cultures. Actually, the presence of wild type strain neither improved nor impaired *pho4* susceptibility to RNS.

### The *pho4* Mutant Has a Low Level of Colonization in a Competitive Commensalism Model

**FIGURE 6:**
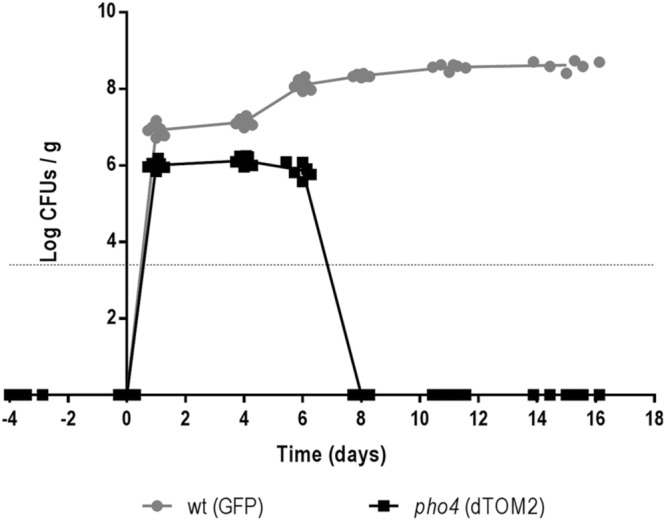
**The lack of Pho4 impaired intestinal colonization in a competitive model**. Wild type and *pho4* mutant tagged with GFP and dTOM2 respectively were mixed and inoculated in antibiotic treated C57BL/6 mice. Colonization levels were followed on time by counting CFUs from stools from six mice.

The relevance of Pho4 in a competitive commensalism model was analyzed. For this purpose an equilibrated mix of labeled wild type/*pho4* mutant strains was inoculated intragastrically in C57BL/6 mice. Gut colonization was followed on time by plating and counting *C. albicans* CFUs from stools. Wild type strain reached colonization level of ∼7 log units per g of stools while the *pho4* mutant only reached around 6 log units per g of stools. In addition, *pho4*-dTOM2 cells were not detected 8 days after inoculation (**Figure 6**). Given that the limit of detection of *C. albicans* cells in stools using our approach is around 10^3-4^/g stools, we discarded that residual cells could still be present in mice; for this purpose, mice were euthanized at the end of the experiment (20 days) and the intestinal content was analyzed; however, no *pho4*-dTOM2 cells were detected along the murine gastrointestinal tract.

**FIGURE 7:**
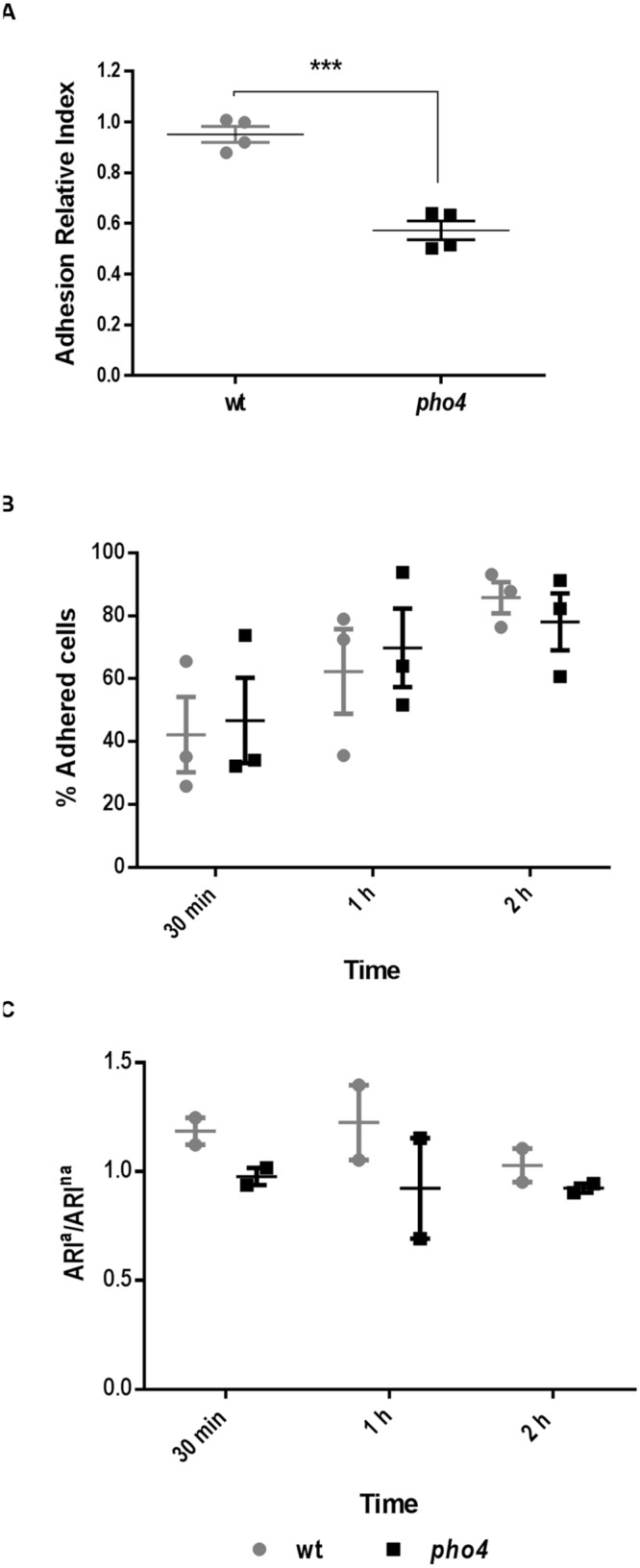
**Pho4 is relevant for adhesion to the intestinal mucose. (A)** Adhesion to intestinal mucosa was assayed using a SFY87-GFP/SFY87- dTOM2 tagged strains as internal control and SFY87-RFP and *pho4*-dTOM2 strains as samples. *C. albicans* mix was allowed to adhere to the murine gut mucosa for 150 min, then the Adhesion Relative Index was calculated by dividing the percentage of adhered cells from (SFY87 or *pho4*)-dTOM2 strains, recovered after 150 min of interaction with gut mucosa, by their percentage in the *inoculum*. Each point represents an individual assay. Unpaired *t*-test displayed a *p*-value < 0.001 ^∗∗∗^ (*p* = 0.0002). **(B)** Adherence to plastic was performed adding wild type and *pho4* single culture to plastic multi well plates. The graph shows the mean of percentage of adhered cells to plastic as function on time of three independent experiments each containing two replicates ± SEM. **(C)** Adherence to plastic of labeled mixed cultures was determined as Adherence Relative Index of adhered cells versus Adherence Relative Index of non-adhered cells (ARI^a^/ARI^na^). The graph shows the mean of two duplicate experiments ± SEM.

As a defective colonization could be dependent on reduced adherence to the intestinal mucosa, we tested this effect in a competitive *ex vivo* assay. In this study a similar proportion of wild type and mutant labeled strains were allowed to adhere to the gut mucosa for 2.5 h. Then, non-adhered cells were removed and adhered cells were spread on SD chloramphenicol plates to quantify the relative proportions of each strain. The Adhesion Relative Index was significantly higher (*p* = 0.0002) in the wild type strain (1) compared to *pho4* mutant (0.6) (**Figure 7A**). These results suggest that Pho4 is important for the cells to adhere to the intestinal mucosa and to establish as gastrointestinal commensal in mice in a competitive model.

Adhesion to plastic using cell cultures 24-well plates was also performed. First, single cultures were tested and percentage of adhesion was followed on time. The adhesion percentage increased in function of time in both wild type and *pho4* mutant strains (**Figure 7B**). No significant differences were detected between strains by a standard *t*-test. Then, labeled mixed cultures were analyzed and the wt-GFP/*pho4*-dTOM2 ration was compared to the wt-GFP/wt-dTOM2 control ratio (**Figure 7C**). Data, expressed as ratio of Adhesion Relative Index of adhered versus non-adhered cells, showed no significant differences between wild type and the *pho4* mutant regarding adhesion to plastic surfaces. This experiment cannot be considered as competitive adhesion assays since the cell number was low enough to allow all cells to adhere to the plastic surface but permits the simultaneous analysis of both strains in the same well and, therefore, under the same experimental conditions. Our results therefore indicate that Pho4 is dispensable for *C. albicans* cells to adhere to plastic under our experimental conditions.

## Discussion

*Candida albicans* is an opportunistic pathogen able to inhabit different niches within the host. Its ability to sense and adapt to diverse environments makes *C. albicans* a versatile and difficult to treat pathogen. Different approaches have been used to identify virulence factors that may influence the interaction or ability to colonize specific host niches (Costa et al., 2013; Amorim-Vaz et al., 2015; Delarze et al., 2015). Our work has made use of a transcription factor knock-out library (Vandeputte et al., 2012) with the aim of identifying elements crucial for the response to stress in this pathogen. A similar screening was previously performed by Homann et al. (2009) to identify any phenotype displayed by these transcription factor mutants. However, we specifically focused our research on oxidative and osmotic stress and also incorporated a different oxidative agent (diamide) which also alters cell wall integrity. The screening allowed us to identify some mutants previously involved in the response to oxidative stress such as *cap1* and *skn7* (Alarco and Raymond, 1999; Singh et al., 2004; recently revised by Dantas Ada et al., 2015), which validates our strategy. Some mutants not previously involved in any of the stresses analyzed were identified (**Table 2**). Among them, only one displayed enhanced susceptibility to both osmotic and oxidative stress. In our screening, a *pho4* mutant showed reduced growth in the presence of NaCl, sorbitol, menadione, diamide and hydrogen peroxide. This defective growth in the presence of stresses was not previously identified by Homann et al. (2009) since this strain already displayed a growth defect on YPD (that is, control plates) and, therefore it was not considered as sensitive to osmotic or oxidative stress. In our hands, the *pho4* mutant grew similarly to the parental SFY87 strain in liquid medium and no differences were detected when growth was followed in time either in complex or defined liquid media (**Supplementary Figure S2**). It is important to note that these *pho4* mutants were generated in a different way in a different background (SN152 and BWP17) which can explain the different behavior. The parental strain used in the present work, SFY87 is derived from BWP17. This strain has a partial heterozygous deletion on chromosome 5 that was inherited from RM1000, while SN152 strain, used by Homann et al. (2009), has two wild-type copies of chromosome 5. In addition, the strategy used to generate the transcription factor mutants was different. Homann et al. (2009) used long sequences flanking ORFs to address gene disruption. However, the collection used in the present work was made by TIGR transposon that allow random mutagenesis by transposon insertion (Dhamgaye et al., 2012). The re-integration of the *PHO4* open reading frame in the *pho4* mutant genome reverts the susceptibility to osmotic and oxidative stress indicating that the observed phenotypes are, in fact, due to the lack of the *PHO4* gene. So, the transcription factor Pho4 is required to adapt and/or tolerate both osmotic and oxidative stress.

We show here that *pho4* mutants also displayed an enhanced susceptibility to nitrosative stress, another kind of stress relevant for phagocytes to attack pathogens (Rementería et al., 1995; Brown et al., 2009). In spite of the susceptibility displayed by *pho4* mutants to all the stresses analyzed, cells were not sensitive to peritoneal macrophages or more resistant to the macrophages RAW264.7 cell line. How can this discrepancy be explained? A quantification of the nitrosative stress using the *YHB1* promoter fused to the luciferase gene reporter from *Renilla reniformis* (RLUC) showed that *C. albicans* strains sensed lower nitrosative stress in the presence of cell lines HL-60 or RAW 264.7 than in the presence of 2 mM SIN-1 or 0.6 mM GSNO (Arana et al., 2007). Similarly, the oxidative stress perceived by *C. albicans* in the presence of 10 mM H_2_O_2_, 5 mM diamide, or 5 mM menadione was significantly higher compared to the oxidative stress sensed in the presence of HL-60 or RAW 264.7 cell lines. In those experiments, oxidative stress was quantified using the promoter of *TRR1* fused to RLUC (Arana et al., 2007). These observations indicate that the concentrations of oxidants or nitric oxide used *in vitro* are clearly higher that the ROS and RNS generated by innate immune cells, or at least, by certain phagocyte cell lines. The use of high amount of stress mediators allows us to identify mutants defective in the response/adaptation to those agents. Nevertheless, the defective response might not be relevant *in vivo* as our results suggest or require an adaptive immune response.

In order to better understand the complexity of a live system such a fungal cell, *in vitro* experimental studies should approach to physiological situations. Since *C. albicans* is a commensal in the intestinal tract of human beings where oxygen availability is reduced (Erecinska and Silver, 2001) and often contain increased levels of CO_2_ (Klengel et al., 2005; Dubin and Estenssoro, 2008), the susceptibility of the *pho4* mutant to different stresses was tested upon different atmospheric conditions trying to mimic those physiological niches. The behavior of the *pho4* mutant depended on the environmental O_2_/CO_2_ availability. These data suggest that Pho4 is not relevant when *C. albicans* cells grow in the presence of CO_2_ or when O_2_ is not available in the surrounding environment. These observations support the absence of defects in host–pathogen interaction models displayed by the *pho4* mutant and reinforce the use of experimental conditions closer to natural/physiological situation. In fact, opportunistic fungal pathogens are exposed to oxygen-limited or hypoxic microenvironments during infection and the ability to adapt to microenvironment is crucial for pathogenesis (revised by Grahl et al., 2012).

With the purpose of deeper analysis the virulence factors of *C. albicans*, different experimental models have been used to analyze the virulence factors of *C. albicans* (revised by Chamilos et al., 2007; Koh, 2013). Classical assays of systemic infection in mice have been complemented with virulence studies using invertebrates such as *Caenorhabditis elegans* or *G. mellonella*. The virulence of the *pho4* mutant was analyzed using the *G. mellonella* model. No significant differences were observed between the wild type and *pho4* mutant. Previously, Romanowski et al. (2012) analyzed the virulence of *pho4* mutants using a phosphate depletion model in *C. elegans* and a slight increase in the virulence of *pho4* cells was detected. Here, we report an increased resistance of *pho4* cells to phagocytes using the murine macrophages cell line RAW264.7 but no differences were observed when others phagocytes were analyzed. Cell line RAW264.7 resembles macrophages with a reduced microbicide arsenal. This fact reinforces the lower candidacide effect displayed by RAW264.7 compared to peritoneal macrophages. Moreover, transcriptional analysis of *C. albicans* internalized by murine macrophages displayed a nutrient-poor environment (Lorenz et al., 2004). It is possible that the *pho4* mutant is not actively growing in the nutrient-poor phagolysosome and consequently would be more resistant to antifungal mechanisms than depend on actively growing cells. Our results, together with previous studies in *C. elegans*, show indeed that Pho4 is not required for virulence and does not significantly affects *C. albicans* virulence.

Non-vertebrates models are useful but very different to the situations normally encountered in a mammalian host. For this reason commensalism, models have been developed to study the commensal state of *C. albicans* (Koh, 2013; Prieto et al., 2014, 2016). These models are closer to the physiological situation compared to mouse systemic models or models using invertebrates. Moreover, commensalism models facilitate the analysis of the commensal to pathogen switch (Koh, 2013). The study of *pho4* mutants in a commensalism murine model showed that this transcription factor was not relevant for *C. albicans* to establish as part of the intestinal microbiota. Nevertheless, *pho4* mutants display defects in competitive assays, suggesting fitness alterations. These fitness alterations were evidenced when a *pho4* mutant was grown in the presence of its parental wild type strain, either in *in vitro* or *in vivo* experiments. This alteration entails a disadvantage that affects the capability to establish as commensal as well as competitive adhesion assays. This fact explains why *pho4* cells reached lower colonization levels and disappear from the intestine in competitive commensalism studies with its parental strain. The loss of the *pho4* mutant as part of mycobiota may be due to its lower adherence to the intestinal mucosa and/or its metabolic and fitness defects.

Phosphate is an essential nutrient which homeostasis is important for cell viability. The transcription factor Pho4 plays a major role in phosphate metabolism and tolerance to inorganic arsenic compounds (Romanowski et al., 2012; Bishop et al., 2013; Urrialde et al., 2015). In the present work we show that Pho4 is also involved in the response to osmotic, oxidative and nitrosative stresses. This role was evidenced in *in vitro* assays and was dependent on the O_2_/CO_2_ concentration suggesting its important role in metabolism and fitness. Although no clear role in virulence can be assigned to Pho4, the lack of this transcription factor renders cells unable to colonize the intestinal tract in a competitive assay. It is possible that Pho4 controls the transcription of adhesins, impairing colonization or phosphate acquisition which could be crucial for survival in the murine gut. Nevertheless, our observations suggest that Pho4 plays a major role in metabolism being crucial to compete not only in the intestinal tract but also in standard liquid culture media.

## Author Contributions

VU, DP: experimental work and design; JP supervisor and written; RA-M experimental design, supervisor, and written.

## Conflict of Interest Statement

The authors declare that the research was conducted in the absence of any commercial or financial relationships that could be construed as a potential conflict of interest.

## Footnotes

1 http://www.fgsc.net

## Supplementary Material

FIGURE S1 **Mutants defective in transcription factor identified in the screening were spotted on YPD plates supplemented with the indicated compounds and incubated at 37°C for 48 h.** Each mutant was spotted just behind its parental strain. Only one compound concentration is shown.

Figure S2 **Growth curves of *pho4* and wild type strains.** *C. albicans* growth was quantified at A600 nm and represented as a function of time. **(A)** Defined medium (SD) and **(B)** rich medium (YPD). Linear regression analyses of the log phase revealed no significant differences in the growth rate between strains (*p* = 0.325 in SD and *p* = 0.3384 in YPD).

Figure S3 **Percentage of viability of *Candida* strains in the presence of HL-60 cells differentiated to PMNs.** Student’s two-tailed unpaired *t*-test was performed to compare two groups.

## Abbreviations

GSNO: S-nitrosoglutathione
MD: menadione
P_i_: inorganic phosphate
PMN: polymorphonuclear
PolyP: polyphosphates
RNS: reactive nitrogen species
ROS: reactive oxygen species
