# Supplementary figures and images for: The Candida albicans Pho4 Transcription Factor Mediates Susceptibility to Stress and Influences Fitness in a Mouse Commensalism Model
Source: Front Microbiol. 2016 Jul 7;7:1062. doi: 10.3389/fmicb.2016.01062 (PMC4935684; doi:10.3389/fmicb.2016.01062)

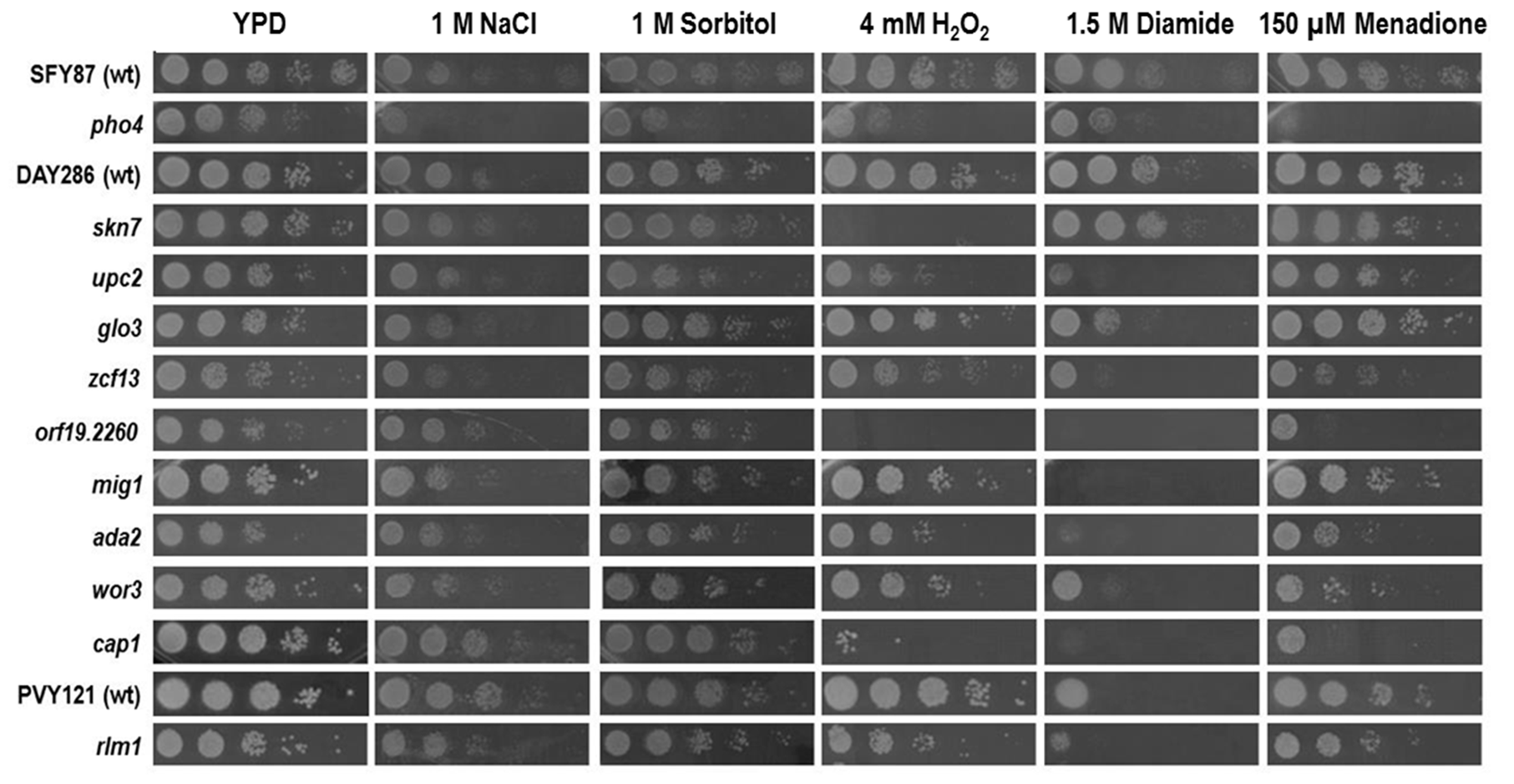

Supplement: FIGURE S1 — Mutants defective in transcription factor identified in the screening were spotted on YPD plates supplemented with the indicated compounds and incubated at 37°C for 48 h. Each mutant was spotted just behind its parental strain. Only one compound concentration is shown. [file Image_1.TIF]

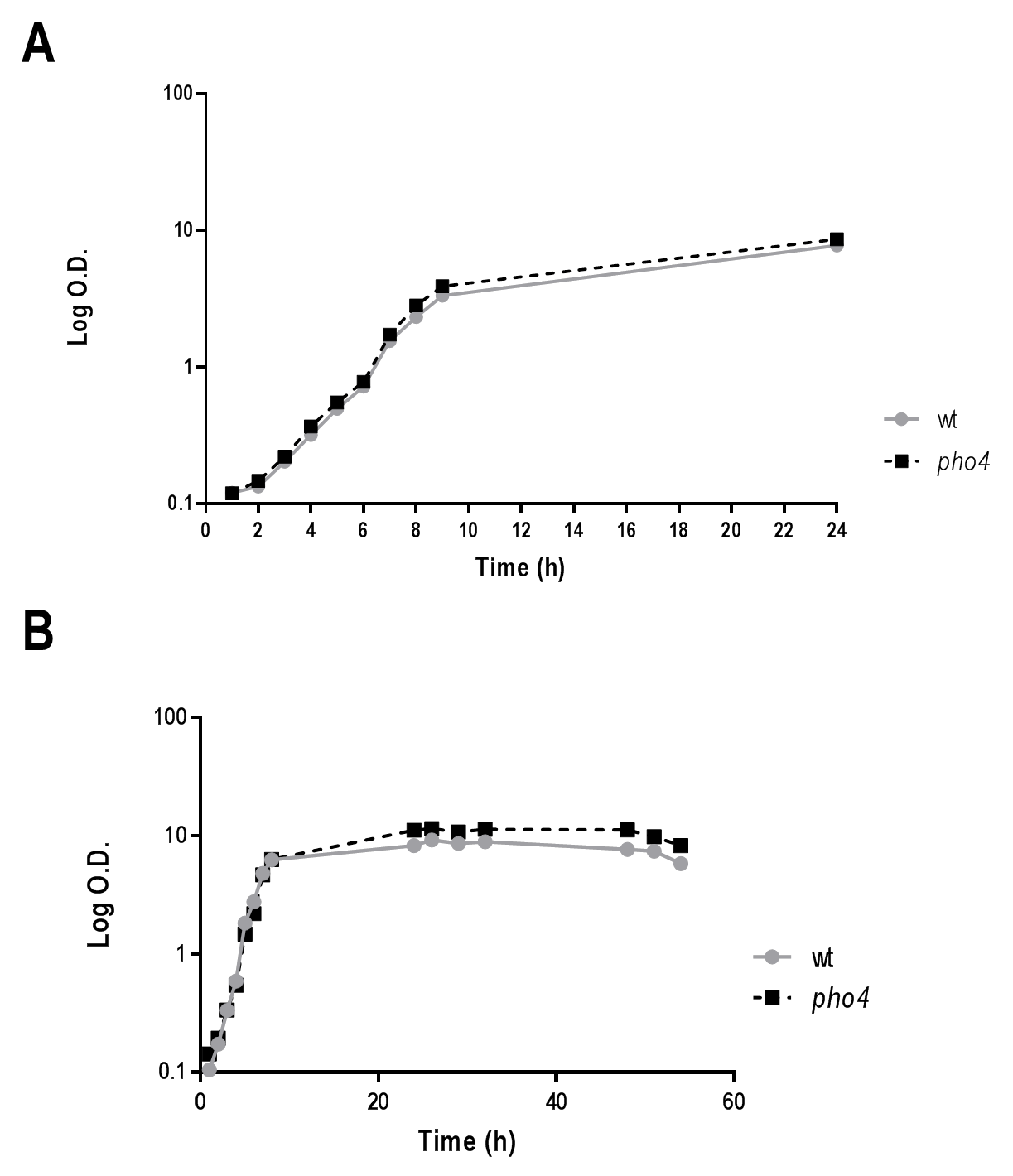

Supplement: Figure S2 — Growth curves of pho4 and wild type strains. C. albicans growth was quantified at A600 nm and represented as a function of time. (A) Defined medium (SD) and (B) rich medium (YPD). Linear regression analyses of the log phase revealed no significant differences in the growth rate between strains (p = 0.325 in SD and p = 0.3384 in YPD). [file Image_2.TIF]

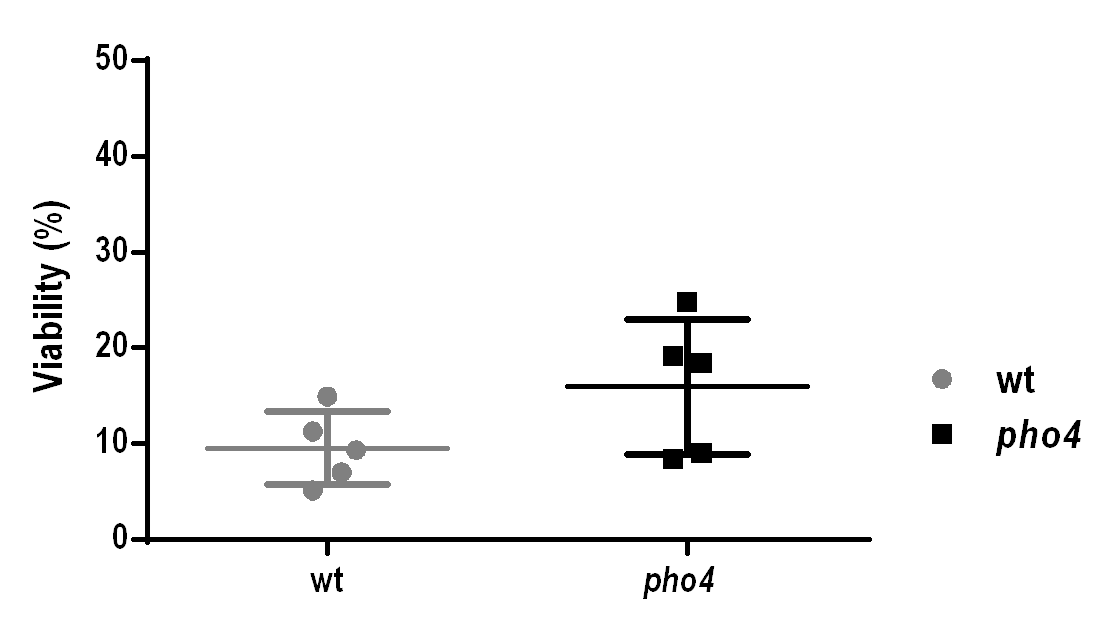

Supplement: Figure S3 — Percentage of viability of Candida strains in the presence of HL-60 cells differentiated to PMNs. Student’s two-tailed unpaired t-test was performed to compare two groups. [file Image_3.TIF]
